# Association of Receipt of Positron Emission Tomography–Computed Tomography With Non–Small Cell Lung Cancer Mortality in the Veterans Affairs Health Care System

**DOI:** 10.1001/jamanetworkopen.2019.15828

**Published:** 2019-11-20

**Authors:** Maya Vella, Craig S. Meyer, Ning Zhang, Beth E. Cohen, Mary A. Whooley, Sunny Wang, Michael D. Hope

**Affiliations:** 1 Department of Radiology and Biomedical Imaging, University of California, San Francisco; 2 Department of Medicine, University of California, San Francisco; 3 San Francisco Veterans Affairs Health Care System, San Francisco, California; 4 Division of Hematology and Oncology, University of California, San Francisco

## Abstract

**Importance:**

Positron emission tomography–computed tomography (PET-CT) has been increasingly used in the management of lung cancer, but its association with survival has not been convincingly documented.

**Objective:**

To examine the association of the use of PET-CT with non–small cell lung cancer (NSCLC) mortality in the US Department of Veterans Affairs (VA) health care system from 2000 to 2013.

**Design, Setting, and Participants:**

This cohort study included 64 103 veterans receiving care in the VA health care system who were diagnosed with incident NSCLC between September 2000 and December 2013. Data analysis took place in October 2018.

**Exposure:**

Use of PET-CT before and/or after diagnosis.

**Main Outcomes and Measures:**

All-cause and NSCLC-specific 5-year mortality; secondary outcome was receipt of stage-appropriate treatment.

**Results:**

A total of 64 103 veterans with the diagnosis of NSCLC were evaluated; 62 838 (98.0%) were men, and 50 584 (78.9%) were white individuals. Among these, 51 844 (80.9%) had a PET-CT performed: 25 735 (40.1%) in the 12 months before diagnosis and 41 242 (64.3%) in the 5 years after diagnosis. Increased PET-CT use (597 of 978 veterans [59.2%] in 2000 vs 3649 of 3915 [93.2%] in 2013) and decreased NSCLC-specific 5-year mortality (879 of 978 veterans [89.9%] in 2000 vs 3226 of 3915 veterans [82.4%] in 2013) were found over time. Increased use of stage-appropriate therapy was also seen over time, from 346 of 978 veterans (35.4%) in 2000 to 2062 of 3915 (52.7%) in 2013 (*P* < .001). Increased PET-CT use was associated with higher–complexity level VA facilities (26 127 veterans [82.3%] at level 1a vs 1289 [75.2%] at level 3 facilities; *P* < .001) and facilities with on-site PET-CT compared with facilities without on-site PET-CT (33 081 [82.2%] vs 17 443 [80.3%]; *P* < .001). Use of PET-CT before diagnosis was associated with increased likelihood of stage-appropriate treatment for all stages of NSCLC (eg, veterans with stage 1 disease: 4837 of 7870 veterans [61.5%] who received PET-CT underwent surgical resection vs 4042 of 7938 veterans [50.9%] who did not receive PET-CT; *P* < .001) and decreased mortality in a risk-adjusted model among all participants and among veterans undergoing stage-appropriate treatment (all-cause mortality: hazard ratio [HR], 0.78; 95% CI, 0.77-0.79; NSCLC-specific mortality: HR, 0.78; 95% CI, 0.76-0.80). Facilities with on-site PET-CT and higher–complexity level facilities were associated with a mortality benefit, with 16% decreased mortality at level 1a vs level 3 facilities (HR, 0.84; 95% CI, 0.80-0.89) and a 3% decrease in all-cause mortality in facilities with on-site PET-CT (HR, 0.97; 95% CI, 0.96-0.99).

**Conclusions:**

In this study, the use of PET-CT among veterans with NSCLC significantly increased from 2000 to 2013, coinciding with decreased 5-year mortality and an increase in stage-appropriate treatment. Variation in use of PET-CT was found, with the highest use at higher–complexity level facilities and those with PET-CT on-site. These facilities were associated with reduced all-cause and NSCLC-specific mortality.

## Key Points

**Question:**

What is the association of the use of positron emission tomography–computed tomography and outcomes for the management of non–small cell lung cancer?

**Findings:**

This cohort study of 64 103 veterans with non–small cell lung cancer in the US Department of Veterans Affairs health care system showed that utilization of positron emission tomography–computed tomography in the 12 months before diagnosis was associated with increased likelihood of stage-appropriate treatment and decreased mortality for veterans with non–small cell lung cancer.

**Meaning:**

Findings suggest that use of positron emission tomography–computed tomography before diagnosis was associated with a higher level of care for veterans with non–small cell lung cancer and that it was associated with decreased mortality.

## Teaser

This cohort study examines the association of the use of positron emission tomography–computed tomography (PET-CT) and non–small cell lung cancer mortality in the US Department of Veterans Affairs health care system from 2000 to 2013.

## Introduction

Lung cancer is the leading cause of cancer-related death, accounting for approximately 25% of all estimated cancer deaths in 2019.^1^ Age and cigarette smoking are the main risk factors for lung cancer.^2^ Given the demographics of the population that receives care in the US Department of Veterans Affairs (VA) health system, specifically the high incidence of smoking, lung cancer is a significant issue for US veterans, accounting for nearly 20% of all cancers among veterans.^3^ It is estimated that 900 000 veterans in the VA health care system meet US Preventive Services Task Force criteria for lung cancer screening, which has led to efforts to implement and refine lung cancer screening and management throughout the VA.^4,5^

Imaging is central to the detection, staging, and monitoring of patients with lung cancer. Lung cancer screening efforts hinge on the use of low-dose computed tomography (CT).^4,6^ While studies have looked at specific regions to see how screening recommendations have been implemented, neither the full extent of implementation nationwide nor the potential ramifications of increased early cancer detection have been reported.^7,8^ Similarly, fluorodeoxyglucose positron emission tomography (PET) and, more recently, PET-CT have been shown to benefit the staging of lung cancer and the evaluation of treatment response, but its use nationwide and its broad effects on management and outcomes have not been studied.^9,10,11,12,13^ The National Comprehensive Cancer Network recommends the use of PET-CT for the staging of lung cancer and for radiation therapy planning.^14,15,16^ However, PET-CT is an expensive resource that requires dedicated expertise and is only present on-site at roughly half of VA facilities nationally.

To better understand the trends in the use of PET-CT for lung cancer management and its association with outcomes, we conducted a retrospective analysis of the association of PET-CT with non–small cell lung cancer (NSCLC) treatment and mortality in a large cohort of veterans in the VA health care system from 2000 to 2013.

## Methods

### Study Population

A total of 64 103 patients diagnosed with incident NSCLC in the VA health care system were identified between September 30, 2000, and December 31, 2013. Patient data, including stage at time of diagnosis as determined by contemporaneous *American Joint Committee on Cancer *staging manuals, were obtained from the VA Central Cancer Registry and merged with electronic medical records in the Corporate Data Warehouse.^17^ We also obtained data from Fee Basis files, which include care that is paid for by the VA but delivered at non-VA facilities. Cases were identified using *International Classification of Diseases for Oncology*, *Third Edition* site codes for bronchus and lung histology codes. We excluded cases if they lacked identification for linkage to VA databases, had missing dates of diagnosis, had missing or discordant staging data, or if cases were diagnosed by autopsy. The University of California, San Francisco, Committee on Human Research and San Francisco VA Medical Center Research and Development Committee approved the study and waived the requirement for informed consent because the study used deidentified data. This study followed the Strengthening the Reporting of Observational Studies in Epidemiology (STROBE) reporting guideline.

### Database Review

Further inclusion criteria was that veterans had at least 1 health care visit at the VA in the 12 months before the date of lung cancer diagnosis to capture those who were using the VA system for their health care rather than those presenting after diagnosis and treatment at another center. The main outcomes for analyses were all-cause mortality and NSCLC-specific mortality at 5 years after diagnosis. Dates of death were determined from the VA Vital Status File. Cause of death was obtained from the National Death Index. Patient characteristics, including sex, race, housing status, marital status, smoking status, and rural vs urban location of residence, were obtained from VA patient care files and Cancer Registry data. Race was included in the demographic data to help assess the generalizability of our data to other populations. Comorbidities were determined by searching *International Classification of Diseases, Ninth Revision *(*ICD*-*9*) codes for inpatient hospitalizations or any outpatient encounters in the 12 months before lung cancer diagnosis and quantified using the Charlson Comorbidity Index score.^18^

We searched the Corporate Data Warehouse for *Current Procedural Terminology* (*CPT*) codes for PET-CT in the 12 months before and the 5 years after NSCLC diagnosis, through December 31, 2018. We defined the 3 following indicator PET-CT variables: (1) PET-CT that occurred in the 12 months before diagnosis, (2) PET-CT in the 5 years after diagnosis, or (3) PET-CT any time in the 12 months before or 5 years after diagnosis. The first indicator variable was included to ensure all veterans who underwent PET-CTs associated with their diagnosis of NSCLC were included, as many veterans in the VA system have had a PET-CT performed months prior to the formal coding of their diagnosis of NSCLC. Stage-appropriate treatment was ascertained from Cancer Registry data and *CPT* codes in VA data and defined as surgery or stereotactic radiation for stage I; at least 2 modalities for stage II and III (ie, chemotherapy plus another modality); and receipt of chemotherapy or targeted therapy for stage IV.

### Facility Analyses

Facilities within the VA can be stratified by their complexity level, ranging from level 3 facilities, which have the lowest patient volume and complexity of cases, to level 1 facilities, which have the highest levels of teaching, research, physician subspecialists, and patient case complexity. Level 1 facilities are further subdivided into level 1a to level 1c, with level 1a being the most complex, with the largest volume, most complex patient cases, greatest amount of teaching and research, largest number and breadth of physician specialists, and highest level of intensive care unit facilities (eTable 1 in the Supplement).

### Statistical Analysis

Summary statistics were calculated, including means, medians, and SDs for continuous variables and frequencies and percentages for categorical variables. We compared patient characteristics by each PET-CT variable, evaluating differences using χ^2^ test statistics. The proportion of veterans who died within 5 years of lung cancer diagnosis was calculated for each year of diagnosis. The 5-year point was chosen as a commonly used mortality measurement in oncology and to provide a standard time frame for all veterans included in our cohort. The most recent diagnoses we studied took place in 2013, and the analysis was conducted in October 2018. To prevent immortal time bias in our analysis of the association with mortality among veterans receiving PET-CT in the 5 years after diagnosis, we modeled PET-CT in the years after lung cancer diagnosis as a time-varying covariate, such that veterans were coded as not having received PET-CT until the first PET-CT after NSCLC diagnosis. Use of PET-CT for all cases and separately by stage at presentation were compared annually for each year of diagnosis. Utilization was also evaluated by VA facility complexity level and whether the facility had on-site PET-CT.

All-cause and NSCLC-specific mortality were estimated by each PET-CT variable using Cox regression models for all cases, stratified by stage of diagnosis. Final hazard ratios (HRs) and 95% CIs were adjusted for age at diagnosis, sex, race, marital status, Charlson Comorbidity Index score, smoking status, substance use, year of diagnosis, and receipt of stage-appropriate treatment. Models for all cases were also adjusted for stage at diagnosis. All-cause and NSCLC-specific mortality were also estimated by VA facility complexity and on-site PET-CT. Statistical significance was set a priori with 2-tailed tests at *P* < .05. All analyses were performed using SAS statistical software version 7.1 (SAS Institute).

## Results

### Overall Cohort

A total of 64 103 veterans with the diagnosis of NSCLC were evaluated; 62 838 (98.0%) were men, and 50 584 (78.9%) were white individuals. Among these, 51 844 (80.9%) underwent a PET-CT, 25 735 (40.1%) in the year before diagnosis and 41 242 (64.3%) in the 5 years after diagnosis. Of those, 19 894 of 25 735 (77.3%) underwent PET-CT in the 3 months before recorded diagnosis and 28 253 of 41 242 (68.5%) in the 3 months after recorded diagnosis. Demographic characteristics of veterans with NSCLC by use of PET-CT are described in eTable 2 in the Supplement. The veterans who received PET-CT were more likely than those who did not to be white individuals (41 142 [80.3%] vs 9442 [78.2%]; *P* < .001), younger than 65 years (21 374 [41.2%] vs 3694 [30.1%]; *P* < .001), and have fewer comorbidities, as demonstrated by a lower Charlson Comorbidity Index score (score of 0, 8868 [17.1%] vs 1507 [12.3%]; *P* < .001) (eTable 2 in the Supplement). Patients who received PET-CT were also more likely than those who did not to have a defined NSCLC tumor subtype (adenocarcinoma: 17 970 [34.7%] vs 3460 [28.2%]; *P* < .001; bronchioalveolar carcinoma: 1102 [2.1%] vs 170 [1.4%]; *P* < .001; squamous cell carcinoma: 18 139 [35.0%] vs 3459 [28.2%]; *P* < .001), whereas those who did not receive PET-CT were more likely than those who did to have a pathologic diagnosis of carcinoma not otherwise specified (382 [3.1%] vs 1300 [2.5%]; *P* < .001) or other non–small cell variant (4788 [39.1%] vs 13 333 [25.7%]; *P* < .001) (eTable 2 in the Supplement).

The number of NSCLC diagnoses per year showed a gradual increase, with a peak in 2009 of 5854 new diagnoses, and then a slow decline to 3915 new diagnoses in 2013 (Figure). The 5-year all-cause mortality rate showed a steady decline over this period, from 879 of 978 veterans (89.9%) in 2000 to 3226 of 3915 (82.4%) in 2013.

**Figure.**
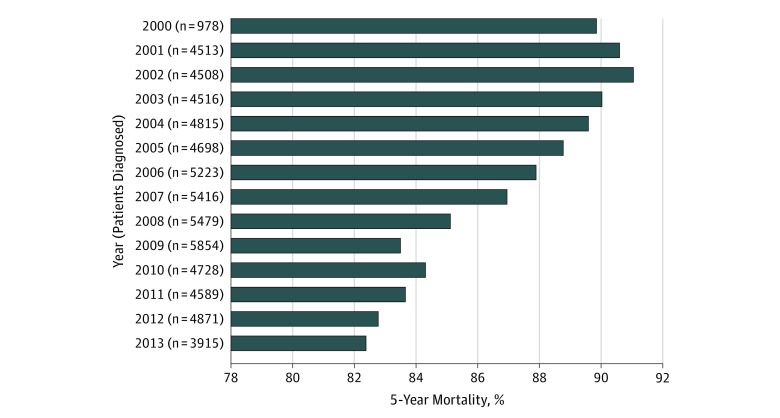
Lung Cancer Diagnoses and Deaths Within 5 Years of Diagnosis

### Subgroup Analyses

A clear trend toward increased PET-CT use was found over time, with 597 of 978 veterans (59.2%) receiving PET-CT in 2000 vs 3649 of 3915 (93.2%) in 2013 (eTable 3 in the Supplement). This increase in use was seen across all stages of NSCLC, with PET-CT being performed more frequently in veterans with lower-stage disease (ie, stages I and II) compared with veterans with higher-stage disease (ie, stages III and IV) during the entire study period (eg, 2000: 201 of 281 [71.5%] vs 378 of 697 [54.2%]; 2013: 1325 of 1369 [96.8%] vs 2324 of 2546 [91.3%]). This trend in use was most clearly borne out in veterans with NSCLC receiving PET-CTs in the 5 years after their diagnosis, with an increase from 387 (39.6%) in 2000 compared with 3122 (79.7%) in 2013. A clear increase in PET-CT use was also found for veterans in the year before diagnosis, ie, 259 veterans (26.5%) in 2000 compared with 1834 (46.8%) in 2013.

### Facility Analyses

Analysis of PET-CT use stratified by VA facility complexity level demonstrated increased PET-CT use at higher–complexity level VA facilities; 26 127 veterans (82.3%) who were treated at level 1a facilities received PET-CTs vs 1289 (75.2%) at level 3 facilities (*P* < .001) (Table 1). There was also higher PET-CT use by veterans receiving care at VA facilities with on-site PET-CT compared with those receiving care at facilities with no on-site PET-CT, albeit to a lesser degree (33 081 [82.2%] vs 17 443 [80.3%], *P* < .001) (Table 1).

**Table 1.** Use of PET-CT by Facility Complexity Level and On-site PET-CT

Facility Characteristic	PET-CT Performed, No. (%)^a^			
	Never	Any	Within 12 mo Before Diagnosis	Within 5 y After Diagnosis
Complexity level^b^				
1a	5603 (17.7)	26 127 (82.3)	13 114 (41.3)	21 222 (66.9)
1b	2901 (19.4)	12 037 (80.6)	5832 (39.0)	9692 (64.9)
1c	1821 (17.9)	8344 (82.1)	4501 (44.3)	6414 (63.1)
2	698 (20.4)	2727 (79.6)	1270 (37.0)	2164 (63.2)
3	426 (24.8)	1289 (75.2)	498 (29.0)	1015 (59.2)
On-site PET-CT				
Yes	7162 (17.8)	33 081 (82.2)	16 769 (41.7)	26 619 (66.1)
No	4287 (19.8)	17 443 (80.2)	8446 (38.9)	13 888 (63.9)

Abbreviation: PET-CT, positron emission tomography–computed tomography.

^a^ *P* < .001 for comparisons of each category with its inverse (eg, PET-CT performed within 12 months before diagnosis vs PET-CT not performed within 12 months before diagnosis).

^b^ Complexity level is based on patient volume, presence of subspecialists, beds, and levels of care, among other characteristics. Full descriptions can be found in eTable 1 in the Supplement.

### Stage-Appropriate Treatment

The overall rate of stage-appropriate treatment showed a significant increase over time, growing from 35.4% (346 of 978 veterans) in 2000 to 52.7% (2062 of 3915 veterans) in 2013 (*P* < .001) (eFigure in the Supplement). A significant association was found between veterans receiving PET-CT in the 12 months before formal diagnosis and stage-appropriate treatment for NSCLC at all stages (Table 2). For example, among veterans with stage I disease, 4837 of 7870 veterans (61.5%) who received PET-CT underwent surgical resection compared with 4042 of 7938 (50.9%) who did not receive PET-CT (*P* < .001). For veterans with stage IV disease, 4092 of 9046 (45.2%) who received PET-CT underwent chemotherapy compared with 6460 of 18 084 (35.7%) who did not receive PET-CT (*P* < .001).

**Table 2.** Receipt of Stage-Appropriate Treatment by Use of PET-CT in 12 Months Before NSCLC Diagnosis

Variable	No. (%)		*P* Value
	PET-CT Performed (n = 25 735)	PET-CT Not Performed (n = 38 368)	
**Stage I**			
Surgery			
Yes	4837 (61.5)	4042 (50.9)	<.001
No	3033 (38.5)	3896 (49.1)	
Radiation^a^			
Yes	609 (7.7)	392 (4.9)	<.001
No	7261 (92.3)	7546 (95.1)	
**Stages II and III**			
Surgery			
Yes	1662 (18.8)	1615 (13.1)	<.001
No	7157 (81.2)	10 731 (86.9)	
Radiation^b^			
Yes	4691 (53.2)	6177 (50.0)	<.001
No	4128 (46.8)	6169 (50.0)	
Chemotherapy			
Yes	4880 (55.3)	5897 (47.8)	<.001
No	3939 (44.7)	6449 (52.2)	
Chemoradiation			
Yes	6170 (70.0)	7937 (64.3)	<.001
No	2649 (30)	4409 (35.7)	
Modalities^c^			
>1	3923 (44.5)	4576 (37.1)	<.001
≤1	4896 (55.5)	7770 (62.9)	
**Stage IV**			
Chemotherapy			
Yes	4092 (45.2)	6460 (35.7)	<.001
No	4954 (54.8)	11 624 (64.3)	

Abbreviations: NSCLC, non–small cell lung cancer; PET-CT, positron emission tomography–computed tomography.

^a^ Includes stereotactic body radiation therapy only.

^b^ Includes stereotactic body radiation therapy and external beam radiation therapy.

^c^ At least 2 modalities used for treatment, specifically chemotherapy plus another modality.

### Five-Year Mortality

Use of PET-CT performed within 12 months before diagnosis of NSCLC but not within 5 years after diagnosis was associated with decreased mortality (Table 3). Receiving PET-CT before diagnosis was associated with a 22% decrease in all-cause mortality (HR 0.78; 95% CI, 0.77-0.79) and a 22% decrease in NSCLC-specific mortality (HR, 0.78; 95% CI, 0.76-0.80) in a risk-adjusted model. Subgroup analysis of all veterans undergoing stage-appropriate treatment demonstrated a 24% decrease in all-cause mortality in veterans who received PET-CT before diagnosis vs those who did not (HR, 0.76; 95% CI, 0.74-0.78). Receiving PET-CT after diagnosis was associated with a 10% increase in all-cause mortality (HR, 1.10; 95% CI, 1.07-1.12) and a 13% increase in NSCLC-specific mortality (HR, 1.13; 95% CI, 1.10-1.16); these results were predominantly associated with veterans with stage I disease. Facilities with on-site PET-CT and higher complexity levels were associated with a relative decrease in mortality, with a 16% decrease in all-cause mortality at level 1a facilities vs level 3 facilities for all stages (HR, 0.84; 95% CI, 0.80-0.89) and a 3% decrease in all-cause mortality in facilities with on-site PET-CT compared with those without (HR, 0.97; 95% CI, 0.96-0.99) (Table 4).

**Table 3.** Hazard Ratios for 5-Year Mortality by Use of PET-CT Within 12 Months Before Diagnosis and Within 5 Years After Diagnosis^a^

Stage	Hazard Ratio (95% CI)^b^		
	All-Cause Mortality	NSCLC-Specific Mortality	All-Cause Mortality With Stage-Appropriate Treatment
**PET-CT Within 12 mo Before Diagnosis**			
All	0.78 (0.77-0.79)	0.78 (0.76-0.80)	0.76 (0.74-0.78)
I	0.91 (0.87-0.95)	0.89 (0.84-0.93)	0.90 (0.85-0.96)
II	0.85 (0.79-0.92)	0.93 (0.85-1.02)	0.80 (0.71-0.91)
III	0.90 (0.87-0.93)	0.92 (0.89-0.95)	0.89 (0.84-0.94)
IV	0.86 (0.84-0.88)	0.86 (0.83-0.88)	0.91 (0.87-0.95)
**PET-CT Within 5 y After Diagnosis**			
All	1.10 (1.07-1.12)	1.13 (1.10-1.16)	1.41 (1.36-1.46)
I	1.18 (1.12-1.24)	1.26 (1.18-1.34)	1.55 (1.43-1.68)
II	0.88 (0.80-0.97)	0.95 (0.85-1.06)	0.79 (0.64-0.99)
III	0.93 (0.90-0.97)	0.93 (0.86-0.97)	0.83(0.77-0.91)
IV	0.98 (0.97-1.03)	1.01 (0.98-1.04)	0.83 (0.78-0.87)

Abbreviation: NSCLC, non–small cell lung cancer; PET-CT, positron emission tomography–computed tomography.

^a^ Adjusted by age, sex, race, marital status, Charlson Comorbidity Index score, smoking, substance use, year of diagnosis, and stage-appropriate treatment (although not in groups where this variable was specifically selected for).

^b^ Hazard ratios are for comparisons of each category with its inverse (eg, PET-CT performed within 5 years after diagnosis vs PET-CT not performed within 5 years after diagnosis).

**Table 4.** Hazard Ratios for 5-Year All-Cause and NSCLC-Specific Mortality by VA Facility Complexity Level and On-site PET-CT

VA Facility Characteristic	Hazard Ratio (95% CI)				
	All Stages	Stage I	Stage II	Stage III	Stage IV
**5-Year All-Cause Mortality**					
Complexity level^a^					
3	1 [Reference]	1 [Reference]	1 [Reference]	1 [Reference]	1 [Reference]
2	0.92 (0.87-0.98)	0.87 (0.75-1.02)	1.07 (0.84-1.36)	1.02 (0.91-1.15)	1.00 (0.91-1.09)
1c	0.91 (0.86-0.96)	0.82 (0.72-0.95)	1.07 (0.86-1.34)	1.03 (0.93-1.14)	1.01 (0.93-1.09)
1b	0.89 (0.85-0.94)	0.79 (0.69-0.91)	1.06 (0.85-1.31)	1.02 (0.92-1.13)	1.00 (0.93-1.08)
1a	0.84 (0.80-0.89)	0.73 (0.64-0.83)	0.99 (0.81-1.22)	0.99 (0.90-1.09)	0.98 (0.91-1.05)
On-site PET-CT					
No	1 [Reference]	1 [Reference]	1 [Reference]	1 [Reference]	1 [Reference]
Yes	0.97 (0.96-0.99)	0.95 (0.91-0.99)	0.94 (0.87-1.01)	0.97 (0.94-1.01)	0.99 (0.97-1.02)
**5-Year NSCLC-Specific Mortality**					
Complexity level^a^					
3	1 [Reference]	1 [Reference]	1 [Reference]	1 [Reference]	1 [Reference]
2	0.91 (0.85-0.97)	0.85 (0.69-1.03)	1.09 (0.82-1.47)	1.00 (0.88-1.14)	0.97 (0.88-1.07)
1c	0.90 (0.85-0.96)	0.80 (0.67-0.95)	1.18 (0.91-1.54)	1.02 (0.91-1.15)	0.98 (0.90-1.07)
1b	0.89 (0.83-0.94)	0.74 (0.62-0.88)	1.10 (0.85-1.42)	0.99 (0.88-1.07)	0.99 (0.92-1.08)
1a	0.83 (0.79-0.88)	0.67 (0.56-0.79)	1.06 (0.82-1.35)	0.96 (0.87-1.07)	0.96 (0.88-1.03)
On-site PET-CT					
No	1 [Reference]	1 [Reference]	1 [Reference]	1 [Reference]	1 [Reference]
Yes	0.98 (0.96-1.00)	0.94 (0.89-0.99)	0.94 (0.86-1.02)	0.97 (0.93-1.00)	0.99 (0.96-1.02)

Abbreviations: NSCLC, non–small cell lung cancer; PET-CT, positron emission tomography–computed tomography; VA, US Department of Veterans Affairs.

^a^ Complexity level is based on patient volume, presence of subspecialists, beds, and levels of care, among other characteristics. Full descriptions can be found in eTable 1 in the Supplement.

## Discussion

This study in the VA health care system demonstrated that the use of PET-CT in veterans with NSCLC significantly increased from 2000 to 2013, coinciding with a decrease in 5-year mortality and an increase in stage-appropriate treatment. We observed an association of PET-CT use with decreased all-cause and NSCLC-specific mortality and with increased rates of stage-appropriate therapy in veterans who received PET-CT within the 12 months before diagnosis compared with those who did not. Our data suggested that PET-CT before diagnosis was associated with improved management and outcomes for veterans with NSCLC, the leading cause of cancer-related death.

The effect of PET-CT on the management of NSCLC through the staging and monitoring of disease, while recognized as important, has not been fully characterized.^9,10,11,12,13,14,19,20^ Our study period (ie, 2000-2013) offered a unique window for assessing the use of PET-CT and its association with the management of veterans with NSCLC. Whereas CT was well established for the assessment of lung cancer, data on PET-CT had not yet been incorporated into lung cancer staging guidelines: the seventh TNM staging criteria were based on a database from 1990 to 2000 that “predates the widespread and routine use of PET.”^21^ Recognition of the importance of PET-CT in the staging process is underscored by its inclusion as a nonanatomic prognostic factor in the current eighth TNM staging criteria for NSCLC.^22,23^ Our study provided specific data on the association of PET-CT use and mortality, which supports the decision to incorporate PET-CT as a standard of practice in the management of NSCLC.

Improved staging of disease likely contributes to the overall association of PET-CT use within the 12 months before diagnosis with mortality that we reported. Research has shown PET-CT to be a useful tool when staging NSCLC and superior to CT alone for mediastinal nodal staging.^9,10,11,12,13,14,19,20^ It has been proposed that the trend toward increased overall survival in patients with NSCLC may be in part because of more accurate staging provided by PET-CT.^23^ Although stage migration bias is known to account for stage-specific improvements in survival with the incorporation of PET-CT, it does not account for the significant associations with improvement in mortality we reported when looking at all stages of disease combined (Table 3).^24^ Our study supported the claim that PET-CT use is associated with both a significantly increased receipt of stage-appropriate therapy and decreased mortality in veterans who received stage-appropriate therapy. This suggests an association between accurate staging by PET-CT and decreased mortality by way of appropriate therapy.

The associations we reported between PET-CT and decreased mortality are multifactorial. The improved outcomes found at higher–complexity level VA facilities and facilities with on-site PET-CT likely reflect the care of dedicated and specialized teams with more advanced resources, including PET-CT. Beyond easier access to PET-CT, higher–complexity level VA facilities likely benefit from having experts in lung cancer who review scans and make diagnostic and treatment recommendations. For example, interpretation of PET-CT findings can be challenging and lead to false-positive results, especially in areas of the United States with endemic fungal infections.^25^ Some of the associations between PET-CT and decreased mortality we reported in Table 3 may be confounded by treatment bias. Veterans with NSCLC may not receive treatment because of a personal choice to forego treatment; we did not have information available to adjust for this patient preference. They would likely receive neither stage-appropriate treatment nor PET-CT, have earlier mortality, and consequently strengthen the association of PET-CT with decreased mortality. However, the clear associations between receipt of PET-CT before diagnosis and decreased mortality in the subset of veterans who underwent stage-appropriate treatment mitigated this bias. While our study does not have data on the specific reason for PET-CT in the year prior to NSCLC diagnosis, given that most veterans (19 894 of 25 735 [77.3%]) received their PET-CT within 3 months before diagnosis, it is likely that the PET-CT was ordered as part of staging work for suspected lung cancer or to guide biopsy before formal coding of NSCLC diagnosis. The increased mortality we reported associated with receipt of PET-CT within the 5 years after NSCLC diagnosis is predominantly associated with veterans with stage I disease; this association may reflect a subset of veterans with complicated courses after treatment who received PET-CT as part of their management compared with veterans with uncomplicated courses who, per National Comprehensive Cancer Network guidelines, only required CT imaging for follow-up once treatment was completed.^14^

Many other factors contribute to mortality in veterans with NSCLC and presumably played a role in the gradual decrease in 5-year mortality found over our study period. Treatment for NSCLC evolved during this time, with landmark studies on stereotactic radiosurgery and targeted molecular agents for treatment reported in the late 2000s and early 2010s.^26,27,28^ Widespread adoption of these therapies likely did not occur until after our study, although their use may be a factor in explaining the decreased mortality found at higher–complexity level VA facilities; earlier adoption of novel therapies may have occurred at these facilities given their affiliations with teaching and research institutions. More recent advances in immunotherapy for treatment of NSCLC were not reported until after the study period.^29,30,31,32^ Staging criteria for lung cancer changed during our study, with the seventh TNM staging criteria first proposed in 2007. A key difference that came with the updated staging criteria was a change in tumor size cutoffs, which had the effect of downstaging many patients.^21^ In clinical practice, this downstaging was often from IIB to IIA and thus unlikely to have an effect on our analysis of stage-appropriate therapy, as these 2 stages typically undergo similar treatment.^33^ However, the updated size cutoffs did downstage some patients from stage IV to stage IIIB, which would allow a wider range of potential therapies that may have contributed to an overall decrease in mortality.^21,34^ Despite the many potential confounding variables at play, the association between PET-CT use and mortality that we demonstrated exists in the context of a risk-adjusted model, which takes into account age, sex, race, marital status, comorbidities (ie, Charlson Comorbidity Index score), year of diagnosis, and receipt of stage-appropriate treatment. It should be noted that our results applied to cases of confirmed NSCLC, not to a screened population where only a minority have NSCLC, and our results reflected the demographic characteristics of our veteran population, which is predominantly male with a high rate of smoking.

Our data showed that PET-CT utilization before diagnosis was associated with decreased mortality for veterans with NSCLC at all stages of disease. Improved management of disease at higher complexity level VA facilities was especially notable. With the release of the US Preventive Services Task Force recommendations for lung cancer screening in 2014,^4^ the implementation of low-dose CT for screening together with appropriate use of PET-CT may help translate the potential gains in earlier diagnosis of NSCLC into lower mortality rates.^35,36,37^

### Limitations

The observational design of our study carries with it the inherent limitation of not being able to determine causality. While our data showed an association between the trends of increased PET-CT utilization at VA facilities and decreased mortality, we cannot claim a causal relationship between these variables. Additionally, the nature of the veteran population (overwhelmingly white men) limits the generalizability of our data. Further studies are warranted in other populations to determine if our findings are more broadly applicable. However, despite these limitations, we feel that, given the high prevalence of lung cancer in the VA population, our findings are important and clinically relevant.

## Conclusions

Our data suggested that PET-CT before diagnosis was associated with a higher level of care for veterans with NSCLC. Furthermore, receipt of PET-CT before diagnosis was associated with decreased mortality.

## Notes

Supplement. **eFigure. **Rate of Stage-Appropriate Treatment by Year **eTable 1. **VA Facility Complexity Level **eTable 2. **Characteristics of Veterans With NSCLC by Utilization of PET-CT Between 2000 and 2013 **eTable 3. **Utilization of PET/CT by Year and Lung Cancer Stage
