# Supplementary material for: Association of Receipt of Positron Emission Tomography–Computed Tomography With Non–Small Cell Lung Cancer Mortality in the Veterans Affairs Health Care System
Source: JAMA Netw Open. 2019 Nov 20;2(11):e1915828. doi: 10.1001/jamanetworkopen.2019.15828 (PMC6902817; doi:10.1001/jamanetworkopen.2019.15828)
Supplement: Supplement. — eFigure. Rate of Stage-Appropriate Treatment by Year eTable 1. VA Facility Complexity Level eTable 2. Characteristics of Veterans With NSCLC by Utilization of PET-CT Between 2000 and 2013 eTable 3. Utilization of PET/CT by Year and Lung Cancer Stage [file jamanetwopen-2-e1915828-s001.pdf]

Supplementary Online Content

Vella M, Meyer CS, Zhang N, et al. Association of receipt of positron emission tomography–computed tomography with non–small cell lung cancer mortality in the Veterans Affairs health care system. *JAMA Netw Open*. 2019;2(11):e1915828. doi:10.1001/jamanetworkopen.2019.15828

eFigure. Rate of Stage-Appropriate Treatment by Year
eTable 1. VA Facility Complexity Level
eTable 2. Characteristics of Veterans With NSCLC by Utilization of PET-CT Between 2000 and 2013
eTable 3. Utilization of PET/CT by Year and Lung Cancer Stage

This supplementary material has been provided by the authors to give readers additional information about their work.

© 2019 Vella M et al. *JAMA Network Open.*

eFigure. Rate of Stage-Appropriate Treatment by Year*

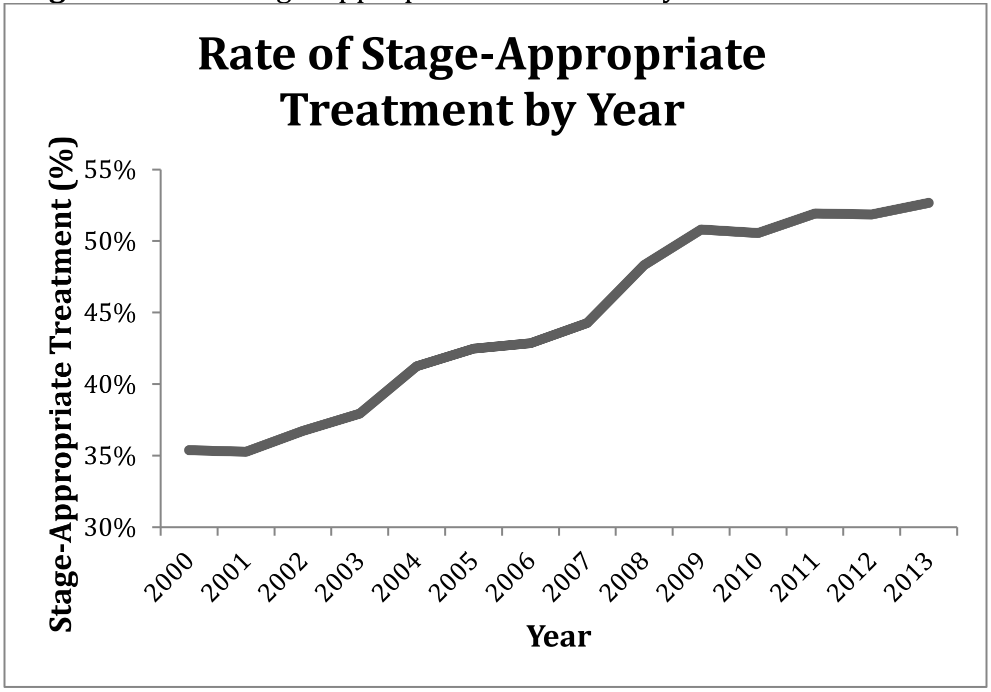

* P < 0.001 for increase in rate of stage-appropriate treatment by year

© 2019 Vella M et al. *JAMA Network Open.*

eTable 1. VA Facility Complexity Level

Complexity level	# VA facilities	Definition
1a	39	• Largest levels of volume, patient risk, teaching and research<br>• Largest number and breadth of physician specialists.<br>• Level 1a facilities contain level 5 Intensive Care Unit (ICU) units.
1b	21	• Very large levels of volume, patient risk, teaching and research<br>• Level 1b facilities contain level 4 and 5 ICU units.
1c	25	• Large levels of volume, patient risk, teaching and research<br>• Level 1c facilities contain level 4 ICU units.
2	24	• Medium patient volume and risk<br>• Some teaching and/or research<br>• Level 2 facilities contain level 3 and 4 ICU units
3	31	• Low levels of patient complexity<br>• Smallest level in terms of volume<br>• Little or no teaching/research<br>• Lowest number of physician specialists per pro-rated person<br>• Level 3 facilities contain level 1 and 2 ICU units.
Excluded	1	
Total	141	

© 2019 Vella M et al. *JAMA Network Open.*

eTable 2. Characteristics of Veterans With NSCLC by Utilization of PET-CT Between 2000 and 2013

Variable		Never Performed (N=12,259)	Any PET/CT (N=51,844)	12 Months Prior (N=25,735)	5 Years After (N=41,242)	P-value*
**Age**	< 65	3,694(30.1%)	21,374(41.2%)	10,467(40.7%)	18,013(43.7%)	<0.001
	65-74	3,698(30.2%)	16,940(32.7%)	8,573(33.3%)	1313,547(32.8%)	
	>74	4,867(39.7%)	13,530(26.1%)	6,695(26.0%)	9,682(23.5%)	
**Sex**	Male	12,043 (98.3%)	50,795(98.0%)	25,194(97.9%)	40,349(97.9%)	0.06
**Race**	White	9,442(78.2%)	41,142(80.3%)	20,454(80.4%)	32,638(80.0%)	<0.001
	Black	2,313(19.2%)	8,599(16.8%)	4,187(16.5%)	6,952(17.0%)	
	Other	319(2.6%)	1,518(3.0%)	788(3.1%)	1,209(3.0%)	
**Married**		5,147(42.6%)	23,425(45.6%)	11,704(45.9%)	18,856(46.1%)	<0.001
**Smoking**	Never	445(4.0%)	1,517(3.1%)	791(3.3%)	1,169(3.0%)	<0.001
	Former	4,693(42.1%)	19,837(40.5%)	9,809(40.6%)	15,836(40.4%)	
	Current	60,06(53.9%)	27,630(56.4%)	13,547(56.1%)	22,149(56.6%)	
**Charlson**	0	1,507(12.3%)	8,868(17.1%)	3,329(12.9%)	7,555(18.3%)	<0.001
**Score**	1-3	4,991(40.7%)	27,815(53.7%)	13,991(54.4%)	22,876(55.5%)	
	>3	5,761(47.0%)	15,161(29.2%)	8,415(32.7%)	10,811(26.2%)	
**Tumor**	Adenocarcinoma	3,460(28.2%)	17,970(34.7%)	9,177(35.7%)	14,751(35.8%)	<0.001
**Type**	Bronchoalveolar	170(1.4%)	1,102(2.1%)	626(2.4%)	953(2.3%)	
	Squamous cell	3,459(28.2%)	18,139(35.0%)	9,310(36.2%)	14,974(36.3%)	
	Other NSCLC	4,788(39.1%)	13,333(25.7%)	6,005(23.3%)	9,535(23.1%)	
	NOS	382(3.1%)	1,300(2.5%)	617(2.4%)	1029(2.5%)	
**Housed**		12,009(98.0%)	50,731(97.9%)	25,148(97.7%)	40,321(97.8%)	0.46
**Region**	Northeast	2,720(22.2%)	10,011(19.3%)	4,715(18.3%)	8,039(19.5%)	<0.001
	West	2,273(18.5%)	9,534(18.4%)	4,691(18.2%)	7,508(18.2%)	
	South	5,299(43.2%)	24,896(48.0%)	12,946(50.3%)	19,692(47.7%)	
	Midwest	1,967(16.0%)	7,403(14.3%)	3,383(13.1%)	6,003(14.6%)	
**Rural**		4,241 (37.3%)	17,360 (40.3%)	8,696 (41.0%)	13,526 (40.0%)	<0.001
**Substance**	Disorder	1,537(12.5%)	7,623(14.7%)	4,034(15.7%)	6,163(14.9%)	<0.001
**HIV/AIDS**		79(0.6%)	355(0.7%)	196(0.8%)	297(0.7%)	0.62
**Hepatitis C**		374(3.1%)	2,418(4.7%)	1,286(5.0%)	2,013(4.9%)	<0.001

* P-values for comparisons of Any PET/CT versus PET/CT Never Performed for given categories.

© 2019 Vella M et al. *JAMA Network Open.*

eTable 3. Utilization of PET/CT by Year and Lung Cancer Stage

Stage/Ye ar	PET/CT Never Performed	Any PET/CT Performed	12 Months Before	5 Years After
**All Stages**				
**2000**	399 (40.8%)	579 (59.2%)	259 (26.5%)	387 (39.6%)
**2001**	1919 (42.5%)	2594 (57.5%)	1304 (28.9%)	1784 (39.5%)
**2002**	1730 (38.4%)	2778 (61.6%)	1389 (30.8%)	1968 (43.7%)
**2003**	1602 (35.5%)	2914 (64.5%)	1423 (31.5%)	2091 (46.3%)
**2004**	1589 (33.0%)	3226 (67.0%)	1550 (32.2%)	2382 (49.5%)
**2005**	1157 (24.6%)	3541 (75.4%)	1611 (34.3%)	2786 (59.3%)
**2006**	766 (14.7%)	4457 (85.3%)	2194 (42.0%)	3430 (65.7%)
**2007**	635 (11.7%)	4781 (88.3%)	2386 (44.1%)	3809 (70.3%)
**2008**	556 (10.1%)	4923 (89.9%)	2434 (44.4%)	4055 (74.0%)
**2009**	542 (9.3%)	5312 (90.7%)	2605 (44.5%)	4432 (75.7%)
**2010**	415 (8.8%)	4313 (91.2%)	2128 (45.0%)	3584 (75.8%)
**2011**	349 (7.6%)	4240 (92.4%)	2248 (49.0%)	3559 (77.6%)
**2012**	334 (6.9%)	4537 (93.1%)	2370 (48.7%)	3853 (79.1%)
**2013**	266 (6.8%)	3649 (93.2%)	1834 (46.8%)	3122 (79.7%)
**Stage 1**				
**2000**	59 (27.4%)	156 (72.6%)	70 (32.6%)	114 (53.0%)
**2001**	320 (30.3%)	736 (69.7%)	383 (36.3%)	550 (52.1%)
**2002**	276 (26.1%)	782 (73.9%)	421 (39.8%)	602 (56.9%)
**2003**	236 (23.2%)	783 (76.8%)	426 (41.8%)	619 (60.7%)
**2004**	201 (19.5%)	830 (80.5%)	432 (41.9%)	681 (66.1%)
**2005**	166 (15.6%)	897 (84.4%)	456 (42.9%)	790 (74.3%)
**2006**	136 (11.3%)	1068 (88.7%)	584 (48.5%)	920 (76.4%)
**2007**	121 (8.9%)	1231 (91.1%)	709 (52.4%)	1090 (80.6%)
**2008**	92 (6.2%)	1383 (93.8%)	801 (54.3%)	1235 (83.7%)
**2009**	94 (5.6%)	1575 (94.4%)	880 (52.7%)	1419 (85.0%)
**2010**	50 (4.3%)	1125 (95.7%)	645 (54.9%)	1013 (86.2%)
**2011**	35 (3.0%)	1121 (97.0%)	701 (60.6%)	1016 (87.9%)
**2012**	38 (2.9%)	1257 (97.1%)	766 (59.2%)	1139 (88.0%)
**2013**	32 (3.1%)	1008 (96.9%)	596 (57.3%)	923 (88.8%)
**Stage 2**				
**2000**	21 (31.8%)	45 (68.2%)	19 (28.8%)	32 (48.5%)
**2001**	84 (33.2%)	169 (66.8%)	88 (34.8%)	129 (51.0%)
**2002**	53 (27.6%)	139 (72.4%)	79 (41.1%)	106 (55.2%)
**2003**	57 (22.5%)	196 (77.5%)	101 (39.9%)	162 (64.0%)
**2004**	46 (18.9%)	198 (81.1%)	91 (37.3%)	166 (68.0%)
**2005**	43 (16.1%)	224 (83.9%)	103 (38.6%)	188 (70.4%)
**2006**	30 (10.7%)	250 (89.3%)	121 (43.2%)	215 (76.8%)
**2007**	17 (5.4%)	295 (94.6%)	143 (45.8%)	250 (80.1%)
**2008**	14 (4.6%)	289 (95.4%)	154 (50.8%)	264 (87.1%)
**2009**	19 (5.6%)	320 (94.4%)	168 (49.6%)	286 (84.4%)
**2010**	28 (6.6%)	395 (93.4%)	215 (50.8%)	354 (83.7%)
**2011**	17 (4.5%)	365 (95.5%)	213 (55.8%)	330 (86.4%)
**2012**	20 (4.5%)	425 (95.5%)	233 (52.4%)	376 (84.5%)
**2013**	12 (3.6%)	317 (96.4%)	171 (52.0%)	287 (87.2%)
**Stage 3**				
**2000**	109 (38.4%)	175 (61.6%)	77 (27.1%)	121 (42.6%)
**2001**	489 (38.1%)	796 (61.9%)	412 (32.1%)	568 (44.2%)
**2002**	436 (33.1%)	880 (66.9%)	422 (32.1%)	641 (48.7%)
**2003**	418 (32.1%)	883 (67.9%)	412 (31.7%)	674 (51.8%)
**2004**	380 (26.9%)	1035 (73.1%)	487 (34.4%)	802 (56.7%)
**2005**	293 (21.0%)	1102 (79.0%)	488 (35.0%)	898 (64.4%)
**2006**	196 (13.0%)	1306 (87.0%)	654 (43.5%)	1050 (69.9%)

© 2019 Vella M et al. *JAMA Network Open.*

**2007**	157 (10.9%)	1281 (89.1%)	662 (46.0%)	1061 (73.8%)
**2008**	139 (9.3%)	1350 (90.7%)	665 (44.7%)	1147 (77.0%)
**2009**	128 (8.4%)	1404 (91.6%)	698 (45.6%)	1206 (78.7%)
**2010**	70 (6.3%)	1050 (93.8%)	505 (45.1%)	911 (81.3%)
**2011**	52 (5.1%)	960 (94.9%)	497 (49.1%)	849(83.9%)
**2012**	57 (5.2%)	1041 (94.8%)	531 (48.4%)	935 (85.2%)
**2013**	32 (3.6%)	858 (96.4%)	410 (46.1%)	771 (86.6%)
**Stage 4**				
**2000**	210 (50.8%)	203 (49.2%)	93 (22.5%)	120 (29.1%)
**2001**	1026 (53.5%)	893 (46.5%)	421 (21.9%)	537 (28.0%)
**2002**	965 (49.7%)	977 (50.3%)	467 (24.0%)	619 (31.9%)
**2003**	891 (45.9%)	1052 (54.1%)	484 (24.9%)	636 (32.7%)
**2004**	962 (45.3%)	1163 (54.7%)	540 (25.4%)	733 (34.5%)
**2005**	655 (33.2%)	1318 (66.8%)	564 (28.6%)	910 (46.1%)
**2006**	404 (18.1%)	1833 (81.9%)	835 (37.3%)	1245 (55.7%)
**2007**	340 (14.7%)	1974 (85.3%)	872 (37.7%)	1408 (60.8%)
**2008**	311 (14.1%)	1901 (85.9%)	814 (36.8%)	1409 (63.7%)
**2009**	301 (13.0%)	2013 (87.0%)	859 (37.1%)	1521 (65.7%)
**2010**	267 (13.3%)	1743 (86.7%)	763 (38.0%)	1306 (65.0%)
**2011**	245 (12.0%)	1794 (88.0%)	837 (41.0%)	1364 (66.9%)
**2012**	219 (10.8%)	1814 (89.2%)	840 (41.3%)	1403 (69.0%)
**2013**	190 (11.5%)	1466 (88.5%)	657 (39.7%)	1141 (68.9%)

* P < 0.0001 for annual utilization of PET/CT by NSCLC stage.
** Percentages are row percentages (total veterans in a given year and stage who received a PET/CT in each category).

© 2019 Vella M et al. *JAMA Network Open.*
